# Highly stable flexible pressure sensors with a quasi-homogeneous composition and interlinked interfaces

**DOI:** 10.1038/s41467-022-29093-y

**Published:** 2022-03-10

**Authors:** Yuan Zhang, Junlong Yang, Xingyu Hou, Gang Li, Liu Wang, Ningning Bai, Minkun Cai, Lingyu Zhao, Yan Wang, Jianming Zhang, Ke Chen, Xiang Wu, Canhui Yang, Yuan Dai, Zhengyou Zhang, Chuan Fei Guo

**Affiliations:** 1 Department of Materials Science and Engineering, Southern University of Science and Technology, Shenzhen, 518055 Guangdong China; 2 College of Polymer Science and Engineering, State Key Laboratory of Polymer Materials Engineering of China, Sichuan University, Chengdu, 610065 China; 3 Tencent Robotics X, Shenzhen, 518000 Guangdong China; 4 School of Materials Science and Engineering, Shenyang University of Technology, Shenyang, 110870 China; 5 Department of Mechanics and Aerospace Engineering, Southern University of Science and Technology, Shenzhen, 518055 Guangdong China

**Keywords:** Electronic devices, Sensors and biosensors

## Abstract

Electronic skins (e-skins) are devices that can respond to mechanical stimuli and enable robots to perceive their surroundings. A great challenge for existing e-skins is that they may easily fail under extreme mechanical conditions due to their multilayered architecture with mechanical mismatch and weak adhesion between the interlayers. Here we report a flexible pressure sensor with tough interfaces enabled by two strategies: quasi-homogeneous composition that ensures mechanical match of interlayers, and interlinked microconed interface that results in a high interfacial toughness of 390 J·m^−2^. The tough interface endows the sensor with exceptional signal stability determined by performing 100,000 cycles of rubbing, and fixing the sensor on a car tread and driving 2.6 km on an asphalt road. The topological interlinks can be further extended to soft robot-sensor integration, enabling a seamless interface between the sensor and robot for highly stable sensing performance during manipulation tasks under complicated mechanical conditions.

## Abstract (web summary)

E-skins often have poor interfaces that lead to unstable performances. Here, authors report e-skins with a quasi-homogeneous composition and bonded micro-structured interfaces, through which both the sensitivity and stability of the devices are improved.

## Introduction

Robots, prosthetics, and other machines gain sensory functions when equipped with electronic skins (e-skins) or flexible pressure sensors^1–6^, which play the role of mechanoreceptors in human skin. Performances of such devices have been significantly improved by introducing new designs such as interfacial microstructures, or doping conductive fillers into the dielectric layer^7–15^. For example, the introduction of microstructures in flexible pressure sensors can improve both the sensitivity by enhancing the compressibility of the dielectric and the response speed of the device by rapidly restoring and releasing energy^13–17^; and adding conductive fillers in dielectric can produce a higher and pressure-dependent dielectric constant and thereby improve signal magnitude^13^. A long-standing challenge for e-skins is their poor stability under harsh and complicated mechanical conditions because of the poor interfaces in the devices. Both the human skin and most e-skins possess multilayer structures; however, they exhibit significantly different levels of mechanical stability. The human skin consists of epidermis, dermis, and subcutaneous fat layers that grow together to have tough interfaces (Supplementary Fig. 1a)^18,19^. Such firm interlocking between the layers allows the skin to survive during manipulation tasks that involve complex mechanical modes such as stretching, torsion, shear, and compression (Supplementary Fig. 1b). By contrast, existing layered e-skins often consist of stacked functional layers (e.g., two electrodes sandwiching a soft dielectric layer) with non-bonded interfaces^16,20,21^. The mechanical stability of such an interface is further undermined when microstructures and air gaps are introduced to improve the sensitivity of the device^22–25^. Another important concern for the weak interfaces in e-skins is the mechanical mismatch between different layers. For example, Young’s moduli of different layers can vary up to five orders of magnitude^16,21,22,26–28^. As a result, delamination or separation of layers readily ensues under complex mechanical deformations, impairing to a large extent the performance of the e-skins and thus the sensory functions of the machine employing the e-skins. Although the integration of interlayers with homogenous composition or fully elastic components that exhibit close rigidity has been used to achieve minimized mechanical mismatch and improved stretchability^29–31^, microstructures and robust interface are not introduced, and thus desired sensing performance and interfacial stability cannot be achieved. Furthermore, integrating e-skins into soft robots or other machines inevitably introduces additional interfaces^32,33^. Such integration likewise suffers from poor interfacial adhesion and mechanical mismatch. It thus remains an urgent necessity to form robust interfaces between different layers for e-skins and for sensor-robot integration.

Here we address the challenges by using a quasi-homogeneous composition for all interlayers and introducing interlinked and microstructured interfaces in a multilayered sensor. The quasi-homogeneous composition made by polydimethylsiloxane-carbon nanotubes (PDMS-CNTs) can avoid the mechanical mismatch between the layers, and the strong topological interlinks between different functional layers can lead to tough and strong interfaces. The sensors consist of a microconed electrode (7 wt% CNTs), a dielectric layer (2 wt% CNTs), and a flat electrode layer (7 wt% CNTs). The microstructured interface with topological interlinks has a high interfacial toughness of 390 J·m^−2^ enabled by two mechanisms: elastic dissipation and discrete rupture of the microstructures. The microcones can be significantly stretched to dissipate energy upon peeling, and the discrete rupture mode stabilizes the interface to prevent catastrophic crack propagation. The CNT doping in the dielectric layer together with the microstructures also boosts the signal intensity of the sensor by a factor of 33. The enhancement of the signal magnitude lies in a composition-structure synergistic effect: the conductive filler enlarges the dielectric constant of the composite upon loading, and the microstructure deformation changes the dielectric-electrode contact area. The tough and strong interface ensures high fidelity of the sensing signal under harsh mechanical conditions: the sensors exhibit adequate signal stability when subjected to repeated rubbing and shear test for at least 10,000 cycles, or when fixated to the tire tread of a running car driven 2.6 km on an asphalt road.

The interlinks can also be applied to the soft robot-sensor interface, and we demonstrate the seamless integration of a soft robot and sensors, both made of PDMS-CNTs composites, for pressure and strain sensing during demanding gripping tasks without any interfacial failure or fatigue. The sensor can identify various stages during the gripping process via the decoupled bimodal signals of capacitance for pressure information. We expect this strategy to be extended to other material systems and other types of sensors for attaining robust interfaces and highly stable signals.

## Results

### Design of the integrated all PDMS-CNTs pressure sensor

**Fig. 1:**
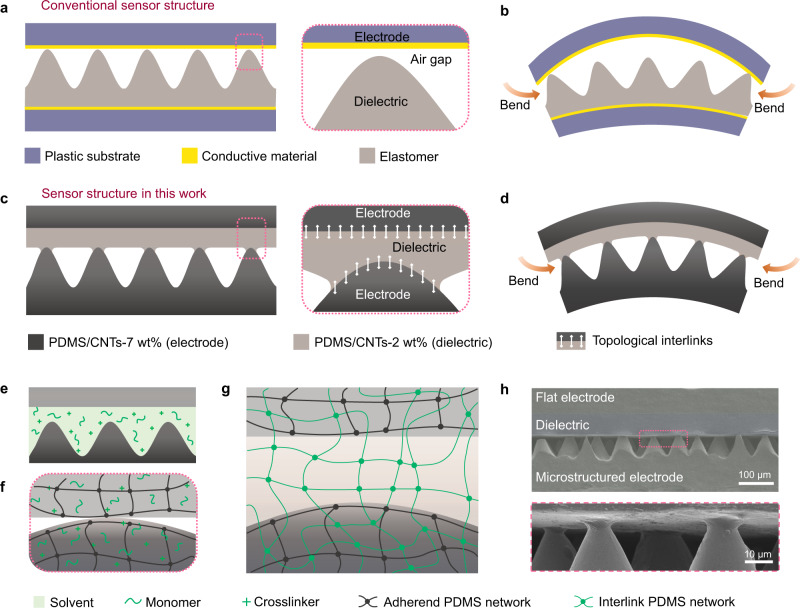
Design of the all PDMS-CNTs-based flexible pressure sensor. **a** Conventional sensors or e-skins have non-bonded interfaces and exhibit significant mechanical mismatch between layers. **b** Delamination readily occurs in conventional sensors upon loading. **c** The all-PDMS-CNTs-based sensor has bonded interfaces and exhibits mechanical match between layers. **d** Interfaces in the all-PDMS-CNTs sensor remains stable under loading. **e** Infiltration by monomers and crosslinkers of PDMS, dissolved in an organic solvent, into the polymer networks of the electrodes and the dielectric layer. **f** The swollen electrodes and dielectric layer are integrated together before curing the infiltrated monomers and crosslinkers. **g** After curing, an interlink PDMS network forms at each interface between an electrode and the dielectric layer, in topological entanglement with the two adherend PDMS networks, resulting in robust electrode-dielectric layer interfaces. **h** Cross-section SEM images of the sensor, showing seamlessly interlinked interfaces between different layers.

In conventional multilayered e-skins, the functional layers are stacked on top of each other without introducing interlayer bonding (Fig. 1a)^16,21–25^, and thus the layers in such devices will easily separate or delaminate when exposed to in-plane compressive stresses or shear stresses (Fig. 1b). In addition, the layers are often made of different materials, which induce remarkable mechanical mismatch^22,26,27,34^. For example, in a capacitive type sensor, the electrodes are mostly metal or indium tin oxide (Young’s modulus *E* ~100  GPa) deposited on plastic substrates (*E* ~ 1 GPa), while the dielectric layer is made of a soft material such as PDMS (*E* ~ 1 MPa)^22,34^. The significant mechanical mismatch between the electrode and the dielectric may cause interfacial failure under large deformations^35,36^.

The devices studied in this work are made of an all-PDMS-CNTs material system to avoid mechanical mismatch, and their functional layers are interlinked (generating a new network that penetrates with the adherend networks) to form tough and strong interfaces (Fig. 1c, d). From top to bottom, the device consists of a flat electrode (7 wt% CNTs, 50 μm thick), a flat dielectric layer (2 wt% CNTs, 120 μm thick), and an electrode that features dense surface microcones (7 wt% CNTs, ~100 μm thick) with an average cone height of ~30 μm and an inter-cone distance of 34 μm^37^. Our selection of the material system of the sensor is based on the characteristics of the composite: the electrodes (with 7 wt% CNTs) are electrically conductive, while the dielectric (with 2 wt% CNTs) has a significant change in dielectric constant upon loading (Supplementary Fig. 2), which will be discussed in details hereinafter. The interlinks between the layers are formed using a procedure developed here. First, the electrodes and the dielectric layer are swollen in a trichloromethane solvent with a PDMS base (5.5 wt%) and a curing agent (0.55 wt%) solute (Fig. 1e). Next, the swollen components are stacked in the order described above, exposed to a pressure of 20 kPa, and cured (Fig. 1f). A new PDMS network is formed, in topological entanglement with preformed PDMS networks, to interlink the layers together (Fig. 1g). With interlinking, all layers are seamlessly integrated at the interfaces, as shown in the scanning electron microscopy (SEM) images in Fig. 1h. Specifically, at the interface between the dielectric layer and the bottom microstructured electrode, the cone tips are found to merge into the dielectric layer (low panel, Fig. 1h).

The interlinking method is specially suitable to bond microstrucrued interface. In the process, monomers and crosslinkers are infiltrated into the preformed polymer networks of the microstructured electrode and the flat dielectric. The microstructures maintain during the swelling process, and a third polymer network forms to interlink the microstructured and the flat surfaces together upon curing. In addition, no adhesives are introduced in the interlinking process, and homogenous composition can thus be achieved.

### Interfacial toughness and strength

**Fig. 2:**
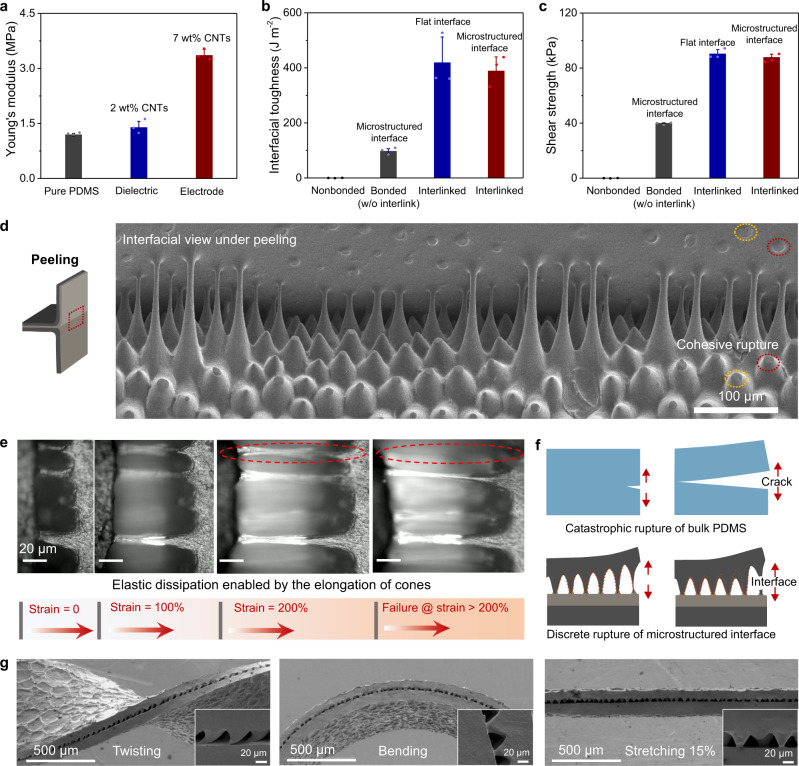
Mechanical properties of the microstructured interface and mechanisms for the tough interface. **a** Young’s moduli of pure PDMS, PDMS-CNTs (2 wt% CNTs) dielectric layer, and PDMS-CNTs (7 wt% CNTs) electrode. Comparisons of **b**, interfacial toughness and **c**, shear strength between a microstructured non-bonded interface, a microstructured bonded interface without interlinks, a flat bonded interface with crosslinks, and a microstructured bonded interface with interlinks. **d** SEM image of the microstructured interface under peeling, showing cohesive ruptures of the microcones marked by dashed ellipses. A schematic illustration of the peeling test is shown at left. **e** Sequential optical images of the microconed interface during in-situ stretching, showing a rupture strain of >200% that enables significant energy dissipation. **f** Schematic illustrations of brittle rupture of bulk PDMS and discrete rupture of a microstructured interface. **g** SEM images of the sensor under (from left) twisting, bending, and stretching, showing stable bonding between the microcones and the dielectric layer.

Our design with a quasi-homogeneous composition of the whole sensor and interlinked interfaces enables mechanical match between the functional layers as well as strong and tough interfaces. In our devices, all the layers exhibit similar mechanical properties because the whole system is based on a PDMS matrix doped with small amounts of CNTs. Figure 2a shows that the Young’s moduli of pure PDMS, PDMS-CNTs composites doped with 2 wt% and 7 wt% CNTs are 1.2, 1.4, and 3.4 MPa, respectively. Although the CNTs doping leads to increased Young’s modulus of the composite, the small difference can hardly cause mechanical mismatch. Such a mechanical match cannot be achieved in other sensor designs that include a soft dielectric layer and metal- or plastic-based electrodes.

We also measured the toughness and shear strength of all interfaces in the device. The flat interface between the top electrode (which is flat) and the dielectric layer has an interfacial toughness of 420 J·m^−2^ and a shear strength of 90 kPa, while the microstructured interface, although containing abundant voids and pores, exhibits an interfacial toughness of 390 J·m^−2^ and a shear strength of 88 kPa, as shown in Fig. 2b, c and Supplementary Fig. 3. Although the interlinked microconed interface has a slightly lower interfacial toughness than the interlinked flat interface that makes the sensor fail at the cones, the microstructured interface will contribute to improved sensing properties while being much tougher than the non-interlinked one. The device’s topological interlinks are crucial to the high interfacial toughness and shear strength. For example, when applying a thin (few microns) liquid layer of a PDMS base and a curing agent (10:1 in weight ratio) as an adhesive between the non-swollen microconed electrode and dielectric layer, because the PDMS precursor is viscous and barely infiltrates into the preformed PDMS networks, the cured interface exhibits a much lower interfacial toughness of ~96 J·m^−2^, only 1/4 that of the interlinked interface. Likewise, the shear strength of the non-interlinked interface is only 39 kPa, also much lower than that of the interface with topological interlinks.

### Elastic dissipation mode and discrete rupture mode

Such a high interfacial toughness is attributed to its significant elastic dissipation and the discrete rupture mode of the cones. First, the strong adhesion of the cone-dielectric interface and the large stretchability of the cones enable high elastic dissipation. On one hand, the cones can be significantly elongated to a large strain (~200%, Fig. 2d, e) to dissipate energy. We ascribe such a large stretchability to the fact that small-scale structures have far fewer flaws than bulk materials^38,39^. On the other hand, the strong adhesion enabled by the topological interlinks allows the cones to survive under large strains until cohesive ruptures of the cones occurs, as shown by SEM observation of a microstructured interface upon peeling (Fig. 2d and Supplementary Fig. 4).

We conducted a calculation of the interfacial toughness based on the elastic dissipation of individual cones and the result matches well with the experimental value. The interfacial toughness is the energy needed to advance the crack by unit area during peeling, or the energy required to break the cones per unit area. Therefore, interfacial toughness can be expressed as

1 $$\varGamma ={E}_{{cone}}\times N$$

where *E*_*cone*_ is the energy required to break an individual cone, and *N* is the areal density of bonded cones. We estimated that about 6.8 × 10^–7^ J energy (*E*_*cone*_) is dissipated when an individual cone is elongated until it fractures, by taking into account the stress-strain curve of the CNT-doped PDMS and the size of the cones. Considering that the areal density *N* is about 7.9 × 10^8^ m^–2^ (Supplementary Fig. 5) and about 20% cones are not bonded due to their small height (which decreases the interfacial toughness), the interfacial toughness based on our elastic dissipater mode is calculated to be 420 J·m^−2^, which is satisfactorily close to the experimental value of 390 J·m^−2^.

Second, the microcones undergo discrete rupture that can locally stabilize the interface to avoid continuous, catastrophic, brittle failure. Although bulk PDMS is soft and stretchable, it becomes brittle and fractures catastrophically once a crack forms and propagates (Fig. 2f). By contrast, at the microstructured interface, the cones are elongated collectively and fracture one-by-one. When the cones at crack tip fracture, they dissipate the stored strain energy and relax the local strain on their neighbors while the rest of the cones ahead of the crack tip deform more and maintain the overall integrity. We call this failure mode “discrete rupture” (Fig. 2f), and it is similar to the delocalized rupture of a two-dimensional nanomesh electrode on an elastomeric substrate^40^ and splitting the contact mechanism in the detachment of gecko’s foot-hair^41,42^. Because of its elastic dissipation and discrete rupture mode features, the microstructured interface exhibits a high interfacial toughness close to 400 J·m^–2^, which is comparable to the fracture toughness of pure PDMS (Supplementary Fig. 6). Note that the rupture of the interface is contributed by the failure of individual microcones, which are three-dimentional structures. The toughening mechanism introduced by a three-dimensional interface offers an opportunity to significantly improve the stability of the interfaces in sensors and other devices.

The toughness and stability of the microstructure interface were further confirmed by in situ inspection in various mechanical modes, including bending, stretching, and twisting (Fig. 2g). No delamination of layers or interfacial failure is observed for our interlinked sample under any of these conditions. If the interfaces are not bonded, however, delamination will occur during bending (Supplementary Fig. 7). Twisting will generate particularly large shear strain at the interfaces, and our SEM analysis shows that the cones remain firmly bonded to the dielectric layer under a large shear strain of ~0.8 or 45° (Fig. 2g and Supplementary Fig. 8), corresponding to a local shear stress of ~400 kPa at the cone tip. The structural stability under large shear strain is important for practical applications since interfacial failures are mostly caused by shear.

### Sensing properties of the sensor

**Fig. 3:**
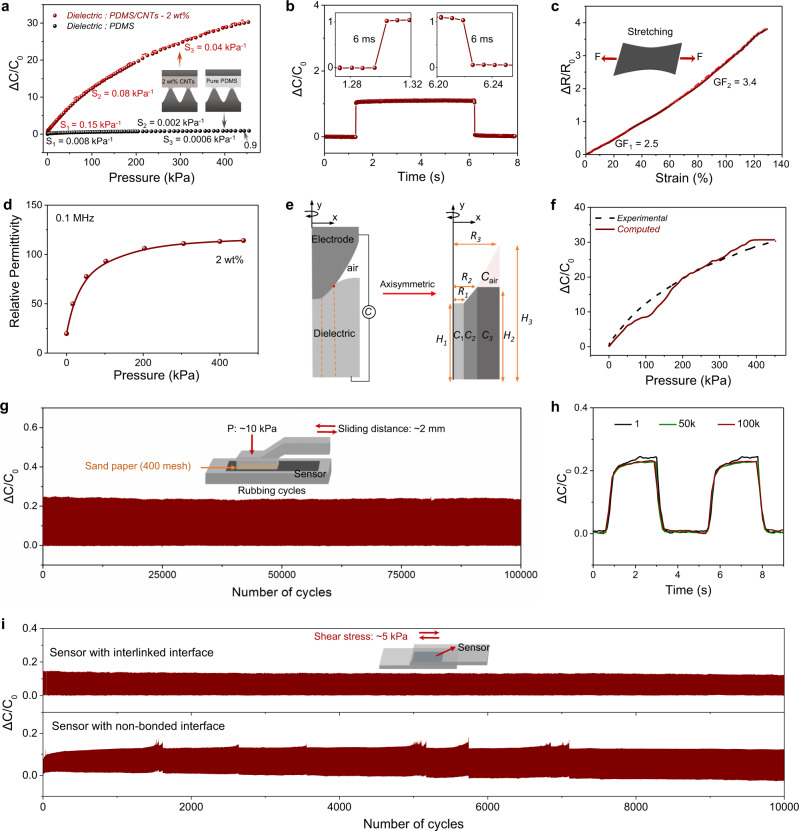
Sensing properties, sensing mechanism, and signal stability of the sensor. **a** Normalized change in capacitance as a function of pressure for a sensor with a dielectric layer of PDMS-CNTs (2 wt%) or pure PDMS. **b** Response and relaxation time. **c** Normalized change in resistance as a function of strain for the flat PDMS-CNTs electrode (7 wt% CNTs), showing a constant gauge factor of 2.5 in the strain range of 0–60%. **d** Relative permittivity of the PDMS-CNTs (2 wt%) dielectric layer as a function of pressure. **e** Model used to calculate the normalized capacitance by considering both the localized microstructure deformation and pressure-dependent permittivity. **f** Calculated normalized capacitance as a function of applied stress using the model shown in panel **e**. **g** Cyclic rubbing test response results. Inset: schematic illustration of the rubbing test. **h** Comparison of the signals at the 1^st^, 50,000^th^, and 100,000^th^ rubbing cycles. **i** Comparison of signal stability over 10,000 shear-release cycles between sensors with bonded (upper panel) and non-bonded (lower panel) interfaces.

The capacitance response of the sensor (10 mm × 10 mm in area) under pressure is shown in Fig. 3a. As a key parameter for pressure sensing, the sensitivity of a capacitive-type sensor is defined as *S* = (Δ*C*/*C*_*0*_)/Δ*P*, where *C*_*0*_ is the initial capacitance before loading and Δ*C* is the change in capacitance with the change in pressure, Δ*P*^43,44^. From Fig. 3a, the sensitivity is 0.15 kPa^−1^ when the pressure is below 47 kPa and it drops to 0.08 kPa^−1^ in the range between 47 and 214 kPa and then to 0.04 kPa^−1^ above 214 kPa up to 450 kPa, as indicated by the dashed lines for *S*_*1*_, *S*_*2*_, and *S*_*3*_, respectively. The normalized change in capacitance (Δ*C*/*C*_*0*_) at 450 kPa is ~30, which is 33 times that of a sensor with a dielectric layer of pure PDMS (Δ*C*/*C*_*0*_~0.90; black circles in Fig. 3a) or with a micro or nanostructured dielectric layer (Δ*C*/*C*_*0*_ ~ 1.4)^44^. The limit of detection (LOD) reflects the minimum pressure that a sensor can resolve at a base pressure of 0 Pa. The LOD of this sensor is determined to be ~0.35 Pa (Supplementary Fig. 9), which is superior to that of human skin (~100 Pa)^45^. The low LOD suggests that the sensor can detect tiny pressure signals or weight down to a few milligrams. Additionally, we studied the sensing performance of four sensors prepared from different batches, and the data indicate a high repeatability (Supplementary Fig. 10).

Response and relaxation speeds are other important sensing parameters that are affected by the viscoelasticity and surface structures of materials^28^. Soft elastomers such as PDMS often exhibit quite low response and relaxation speeds because of their high viscosity and sticky surfaces. We tested the response and relaxation time of our sensor (7 mm × 7 mm in area) by applying, holding, and removing a pressure of 1.1 kPa. Both the response time and the relaxation time were measured to be 6 ms (Fig. 3b), which is actually the temporal resolution of our LCR meter. This hints that the true response and relaxation speeds should be even faster.

We further measured the capacitance-pressure hysteresis loops of the device by loading from 0 to 100 kPa and releasing back to 0 kPa at a loading rate of 0.8 kPa s^−1^, and the results are displayed in Supplementary Fig. 11. The curves of loading and unloading almost overlap completely, indicating that the sensor can precisely detect the pressure in both the loading and the unloading processes.

The PDMS-CNTs electrode (7 wt% CNTs) can also be used as a strain sensor. In the strain range of 0–60%, the flat electrode exhibits a constant gauge factor (GF) of 2.5 (dashed-dotted line for GF_1_ in Fig. 3c). GF is defined as the Δ*R*/(*R*_*0*_·*e*), where Δ*R* is the change in resistance with loading, *R*_*0*_ is the initial resistance before loading, and *e* is the engineering strain. Such a response has high repeatability over 10,000 cycles (Supplementary Figs. 12 and 13). Therefore, our device can be used as a bimodal sensor—providing a capacitance signal for pressure sensing and a resistance signal for strain sensing. The two signals decouple from each other since they are measured from different channels (Supplementary Fig. 14). Furthermore, the sensor can be stretched to ~160% (Supplementary Fig. 15), which is sufficient for most applications in conventional robotic systems and on soft robots. It should be noted that strain leads to a limited contribution on the capacitance signal judged from the experimental results on the capacitance to strain response (Supplementary Fig. 16), which is negligible to the overall capacitance change (Δ*C*/*C*_*0*_ ~30 under a pressure of 450 kPa).

### Sensing mechanism: a synergistic effect of composition-structure design

The doping by CNTs not only determines the function of different layers (electrode or dielectric), but also significantly boosts the signal magnitude of the sensor by over 30 times through a synergetic effect of composition and microstructure design. Figure 3d shows that the doping of CNT (2 wt%) can significantly increase the relative permittivity of the dielectric layer, and the relative permittivity becomes highly pressure-dependent: it increases from 19.8 to 114 as the pressure increases from 0 to 460 kPa. The increased relative permittivity can be explained by the power-law equation based on the percolation theory: $\varepsilon \propto {({f}_{c}-{f}_{{CNTs}})}^{-s}$, where *ε* is the relative permittivity, *f*_c_ is the percolation threshold, and *f*_CNTs_ is the CNTs concentration. A shortened distance of neighboring CNTs upon loading leads to the decrease of *f*_c_ and thereby increased *ε*^46,47^. Without CNT doping, the relative permittivity of a pure PDMS is small and its change upon loading is limited—the maximum relative permittivity is only 3.98 at 450 kPa (Supplementary Fig. 17).

When pressure is applied on the sensor, the compressive stresses, as well as the relative permittivity of the underlying dielectric layer will be dramatically amplified by the cone-tips (Supplementary Fig. 18 and Supplementary Movie 1). It should be noted that the air gap also serves as a dielectric. As such, our sensor consists of many microconed capacitors and a mesh-like air-gapped capacitor connected in parallel (Supplementary Fig. 19).

To elucidate the pressure sensing mechanism, we extract the deformed configurations of the microstructured interface and calculate the capacitance of individual unit by using the simplified electric circuit model as depicted in Fig. 3e. The total capacitance *C* is roughly divided into three parts of dielectric layer denoted as *C*_*1*_, *C*_*2*_, and *C*_*3*_, respectively, and one part of air denoted as ${C}_{{air}}$. The total capacitance can be expressed as: $C\approx {C}_{1}+{C}_{2}+\frac{{C}_{3}{C}_{{air}}}{{C}_{{air}}+{C}_{3}}$, where ${C}_{1}\approx \frac{{\epsilon }_{1}\left(P\right){R}_{1}^{2}\pi }{{H}_{1}}$, ${C}_{2}\approx {\int }_{{R}_{1}}^{{R}_{2}}\frac{{\epsilon }_{2}\left(P\right){\left(x-{R}_{1}\right)}^{2}\pi }{\frac{{H}_{2}-{H}_{1}}{{R}_{2}-{R}_{1}}(x-{R}_{1})+{H}_{1}}{dx}$, ${C}_{3}\approx \frac{{\epsilon }_{3}\left(P\right)\left({R}_{3}^{2}-{R}_{2}^{2}\right)\pi }{{H}_{3}}$, ${C}_{{air}}\approx {\int }_{{R}_{2}}^{{R}_{3}}\frac{{\epsilon }_{0}{\left(x-{R}_{2}\right)}^{2}\pi }{\frac{{H}_{3}-{H}_{2}}{{R}_{3}-{R}_{2}}(x-{R}_{2})}{dx}$. Here ${\epsilon }_{0}$ represents the vacuum permittivity, and ${\epsilon }_{1}\left(P\right)$, ${\epsilon }_{2}\left(P\right)$, ${\epsilon }_{3}\left(P\right)$ are the pressure-dependent permittivity of the dielectric parts (Fig. 3d); *R*_*1*_, *R*_*2*_, and *R*_*3*_ are the radii of circular capacitors *C*_*1*_, *C*_*2*_, and *C*_*3*_, respectively; *H*_*1*_, *H*_*2*_, and *H*_*3*_ are the dielectric thickness of circular capacitors *C*_*1*_, *C*_*2*_, and *C*_*3*_, respectively, and *x* is the distance to the cone axis. Note that pressure distribution inside each part of the dielectric layer is approximated to be uniform because higher stress is only observed in the tip contact zones and parameters *R*_*1*_-*R*_*3*_, *H*_*1*_-*H*_*3*_ vary with the pressure (Supplementary Fig. 18). At different compressive pressures, the contact zone configuration changes, leading to different ${R}_{1},{R}_{2},{R}_{3},\,{H}_{1},{H}_{2},{{{\mbox{and}}}}\;{H}_{3}$. By extracting them from the FEA model, we can calculate the total capacitance *C*. Next, normalized Δ*C/C*_0_ is computed and shown in Fig. 3f, which agrees well with experimental measurement.

Our model indicates that the capacitance change is a synergistic effect of the cone structures (for local stress amplification and change in electrode-dielectric contact) and CNT doping (which enables pressure-dependent relative permittivity). By comparing Fig. 3d, f, we can conclude that the response at the high-pressure region (pressure > 200 kPa) is mainly contributed from the localized microstructure deformation, while the response at low pressures is mainly contributed from the permittivity change enabled by the CNT doping. Without a microstructured electrode, the sensor with CNT-doped dielectric and all flat electrodes exhibits a maximum Δ*C*/*C*_0_ of only 5 (Supplementary Fig. 20), and the pressure response range of the sensor becomes much narrow.

### High stability of signal during cycling

Our sensor exhibits high stability under cyclic loading-unloading. We tested the signal stability of the sensor (10 mm × 20 mm in area) under rubbing and shear conditions, each for at least 10,000 cycles. Figure 3g, h show that when the sensor is rubbed with abrasive paper for 100,000 cycles under a normal pressure of 10 kPa and a reciprocating displacement of 2 mm, no obvious change in signal waveform or amplitude is observed. By contrast, the sensing signal of a commercial flexible pressure sensor is apparently unstable (Supplementary Fig. 21). We also tested the signal stability by applying repeated shear stress of 5 kPa (Fig. 3i and Supplementary Fig. 22) for 10,000 cycles, and likewise no significant change in signal amplitude or mechanical failure was observed. By contrast, control samples with a non-bonded microstructured interface show clear signal drift (low panels, Fig. 3i).

### Applications of the sensor under harsh mechanical conditions

**Fig. 4:**
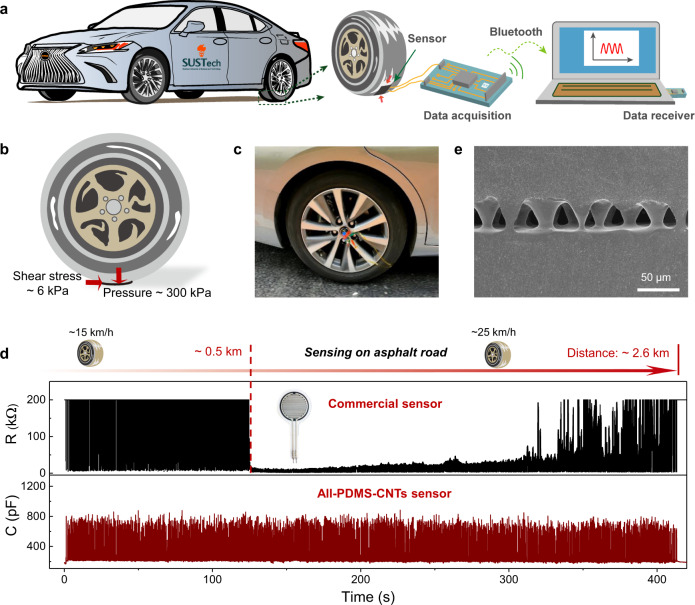
Test of the all PDMS-CNTs sensor and a commercial sensor on a tire tread. **a** Schematic illustration of the testing setup, which consists of a sensor attached to a tire tread, a data acquisition module, and a Bluetooth transmitter for sending test data to a computer. **b** Stress conditions on the sensor. **c** Photograph of the testing system. The sensor is affixed to the tire tread and the data acquisition module and the Bluetooth transmitter are affixed to the wheel hub. **d** Signal stability of the all PDMS-CNTs sensor and the commercial sensor during operation of the car over ~2.6 km on an asphalt road with an average speed of 22 km h^−1^. **e** SEM image of the microstructured interface of the all PDMS-CNTs sensor after testing.

Our design of the material system and the interface enables performances that otherwise cannot be achieved without the advancements made in this work. We tested the stability of our sensor under extreme mechanical conditions, and a running car that generates a high pressure and a large shear stress at its tire tread was used for this purpose. We affixed a sensor (10 mm × 40 mm in area) to the tire tread to test its signal stability during driving, and meanwhile a commercial pressure sensor serving as the control sample was also tested. The capacitance signal was collected using a data acquisition module, and the measured data were transmitted to a computer through Bluetooth (Fig. 4a, and Supplementary Note 1). When the test car is running, the tire tread is loaded with a normal pressure of ~300 kPa and a shear stress of about 6 kPa (Fig. 4b, c), and thus it is a great challenge for a flexible pressure sensor to survive under such complicated mechanical conditions. The calculation of the pressure and the shear stress can be seen in Supplementary Note 2.

We tested the capacitance signal of our sensor and resistance signal of the commercial sensor when the car was in motion over a long distance on an asphalt road. Both sensors performed normally at the beginning of the test (Supplementary Fig. 23). Figure 4d shows that, as the car was driving with an average speed of 22 km·h^−1^, the capacitance signal remained stable over at least 2.6 km (or 1102 rotations). However, the commercial sensor failed after driving over a shorter distance of 0.5 km. We also tested other samples of our sensor under different road conditions and all of them exhibited high stability (Supplementary Movie 2). Note that the non-uniform signal amplitudes of our sensor is caused by the rough surface of the asphalt road containing millimeter and centimeter scale features. The high stability of the signal is in line with the microstructure of the sensor displayed in Fig. 4e, which shows that the cones remain well bonded at the interface without rupture after testing. We ascribe the high stability of our sensor under such harsh mechanical conditions to the quasi-homogeneous material system as well as the interlinked interfaces between different functional layers.

### Soft robot-sensor integration

A requirement for next-generation soft robots is to merge with e-skins to gain sensory functions, such that they can interact with human beings and the environment. Existing sensors and robots, however, are often made of different materials, and thus their integration suffers from the poor sensor-robot interface due to the large difference in mechanical properties. Such integration thus requires complicated designs like embedding the sensor inside the robotic matrix^48^.

**Fig. 5:**
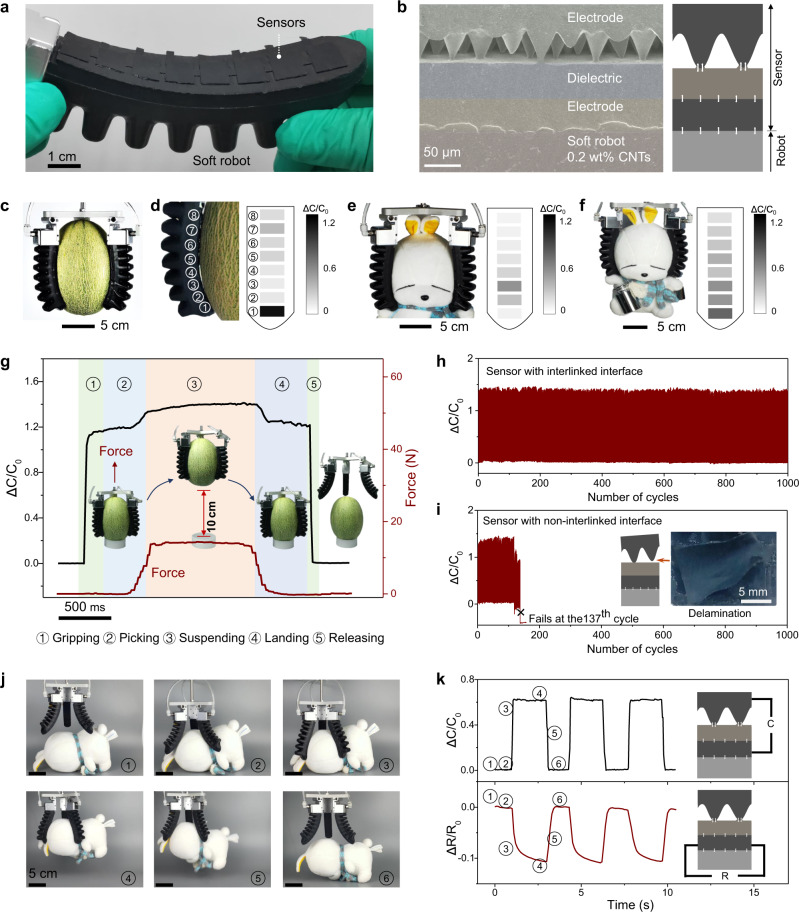
Soft robot-sensor integration. **a** Photograph of a soft robot integrated with a sensor array consisting of eight sensors. **b** Left: SEM image showing the robot-sensor interface and the interfaces in the sensor. Right: schematic illustration of the integrated robot-sensor configuration. **c** Photograph of soft-robot grippers grasping a netted melon. **d** Magnified image showing the contact between the melon and one gripper. The sensors are numbered from 1–8 from bottom to top, and corresponding pressure mapping on the surface of the gripper surface. **e** Photograph of the grippers grasping a stuffed doll and corresponding pressure mapping. **f** Photograph of the grippers grasping a stuffed doll with a weight to have a total mass of 1250 g, and corresponding pressure mapping. **g** Capacitance change evolution of sensor 1 [as marked in panel **d**] as the soft-robot grippers grasp the melon on a table, lift it up to 10 cm, hold it at this height for 1 s, return it to the table, and release it. The photographs illustrate each stage of the manipulation process. The evolutions of the height of the melon and the change in force throughout the process are also plotted. **h** Capacitance change measurement over 1000 cycles of the manipulation stages indicated in panel **g**. **i** Capacitance change measurement of a control sample with a bonded but non-interlinked interface. The sensor fails at the 137^th^ cycle. Inset: schematic illustration of delamination in the control sample and optical image of the delaminated electrode in the control sample. **j** Photographs of soft-robot grippers grasping, lifting, holding, and releasing of a doll. **k** Corresponding capacitance signal for pressure sensing (upper panel) and resistance signal for strain sensing (lower panel) during three cycles of the manipulation shown in **j**. All measured from sensor 1 [as marked in panel **d**]. Upper and lower insets: schematic illustrations of the measuring circuits for the capacitance and resistance signals, respectively.

Here both our sensor and the soft robot are made of PDMS-CNTs composites with interlinked interfaces. Figure 5a shows a photograph of a soft gripper with eight sensors integrated on its surface. The SEM image of a section of the gripper displayed in Fig. 5b shows that the gripper matrix has merged with the bottom electrode of the sensor. Because of the tough interfaces in the robot-sensor system, the soft robots can be used both to grasp objects and to detect the pressure distribution on the gripper surface. Figure 5c–g show grippers being used to grasp a netted melon (weight: 1250 g) and a stuffed doll (weight: 180 g), as well as the corresponding pressure mapping of a gripper surface. Grasping heavy items can generate pressure and shear stress of tens of kilopascals on the gripper surface. When grasping the melon, which is relatively heavy and hard, the gripper surface does not fully conform to the curvature of the melon (Fig. 5d) and the pressure is mainly focused on the bottom of the gripper (Fig. 5d) since the melon tends to slip down and makes more intimate contact with the bottom sensor. By contrast, when grasping a stuffed doll that is softer and lighter (Fig. 5e), the contact elevates to a higher position and a more gradually varied pressure distribution is observed due to its lighter weight, as indicated by the corresponding pressure mapping. Furthermore, when setting a weight on the doll to equalize the weight as the netted melon (1250 g), a gradually varied pressure distribution focused at the bottom of the gripper is observed (Fig. 5f), which combines the features of the previous two cases.

During cyclic manipulation of the melon by the soft robot, the capacitance signal can precisely identify the specific manipulation stage, and it shows high stability over at least 1000 cycles. A force gauge is setup to record the force that lifts the griper during the different manipulation stages (lifting, holding, and landing). Before the melon is lifted, the force is zero. Figure 5g shows the manipulation cycle, including grasping the melon placed on a table, lifting it up to ~10 cm, holding it at this height for ~1 s, returning it to the table, and releasing it. The capacitance signal reflects the different manipulation stages (Fig. 5g and Supplementary Fig. 24). After repeating the process for a total of 1000 cycles, neither a significant change in signal amplitude nor delamination of the sensor was observed (Fig. 5h). The high stability is due to the interlinking of all interfaces in this system. Without this strong topological adhesion, sensors cannot survive under such harsh mechanical conditions. This is shown by repeating the manipulation-cycling experiment with a control sample, in which a thin PDMS layer was used to adhere the microcones and the dielectric layer (without introducing interlinks). In this case, the device delaminates and fails at the 137^th^ cycle (Fig. 5i).

In addition, our devices can serve as bimodal sensors by responding sensitively to both pressure and strain, which are measured from capacitance and resistance signals, respectively. Here, we integrated the sensor on a soft-robot gripper and demonstrated the capacitance and resistance response under a dynamic process of grasping, lifting, holding, and releasing a doll. In the initial state, the gripper is fully open in order to grasp the large item, and a tensile strain is imposed on the sensor. Upon touching and grasping the doll, the capacitance increases sharply, and the resistance decreases due to the reduced strain on the gripper surface. The doll is then lifted and held for ~2 s, and it falls down upon release (Fig. 5j). Accordingly, the capacitance signal remains unchanged during holding, and suddenly decreases to the initial value after release; the resistance signal also shows a relatively stable value when the doll is held and recovers to the original values after release (Fig. 5k).

Moreover, the sensor does not require encapsulation. Encapsulating tactile sensors or e-skins is often conventionally achieved by piling up the device’s functional layers and sealing them with a layer of tape^29,49^. Such sensors work stably under normal compression, but fail under other mechanical modes that generate in-plane shear stresses. Supplementary Fig. 25 shows that in a sensor sealed and affixed to a soft-robot gripper with a thin layer of polyimide tape, the top functional layer buckles upon concave bending due to the much higher rigidity of the tape and the non-bonded interface. As a result, the capacitance signal becomes negative during cycles of bending and release since a thick air gap is generated between the electrode and the CNTs-doped dielectric layer. In comparison, our sensor with tough interfaces does not show any structure damage or signal degradation over bending-release cycling (Supplementary Fig. 23). All these results confirm that our design, which incorporates an all-PDMS-CNTs composition together with interlinked interfaces, is a good selection for sensor-integrated soft robots.

## Discussion

Traditional e-skins designs have mainly focused on the improvement of sensing performance, while few efforts have taken robust interfaces into considerations. Challenges among interfaces stem either from the interlayer debonding/delamination in the sensors, or from the poor fixation of the sensors on robots. Additive manufacturing is expected to be promising for integrated e-skins and for sensor-soft robot integration achieved by multimaterial printing^50^; but this technology is still in its infancy and is currently limited to strain sensors with simple device structures.

Here we still used the traditional multilayered design for sensors and for sensor-soft robot integration, but simply applied a single-material system with tunable functions, together with the interlinking of the layers. Our design minimizes the mechanical mismatch and achieves strong adhesion between different layers in sensors, and between the sensors and a soft robot, while significantly boosting sensing performances. As expected, the tough and strong interfaces remarkably improve the signal fidelity of the sensor under extreme mechanical conditions. Although our strategy of using a single material-system and introducing interlinks between the layers can provide highly stable signal, the maximum sensitivity is relatively low, which might be due to the low conductivity of the electrodes^17,51,52^. This problem can possibly be addressed by using other highly conducting fillers such as Ag nanowires, or by using an ionic material to replace the dielectric layer that help form a supercapacitor at the interface.

In summary, we have developed all-PDMS-CNTs-based sensors with topologically interlinked interfaces, integrated the sensors on soft robots, and demonstrated stable sensing performance under harsh mechanical conditions. By controlling the concentration of CNTs in PDMS, the function of the composite can be tuned to be a dielectric layer or an electrode, while both exhibit similar mechanical properties. The interlinked interface as well as the doping of CNTs in dielectric remarkably boosts the signal amplitude and sensitivity by tens of times. The interlinked interfaces have a high toughness of >390 J·m^−2^, and a shear strength of >88 kPa. As such, the sensor exhibits negligible fatigue when subjected to repeated rubbing and shear test for at least 10,000 cycles, or when attached on a tire tread and run over 2.6 km. The high toughness and strength of the microstructured interfaces stem from the topological interlinks, the elastic dissipation caused by the microcones, and the discrete rupture mode of the cones. The interlinked interface and the all-PDMS-CNTs composition can also be extended to the integration of e-skins on soft robots, and we have shown that e-skins bonded on soft robot grippers exhibit high stability during manipulation tasks under harsh mechanical conditions. Our strategy opens a path for the fabrication of highly robust e-skins with improved sensitivity and response/relaxation speeds, as well as for the integration of sensors and soft robotics.

## Methods

### Preparation of all PDMS-CNTs functional layers

CNTs (0.77 g, purity: 95%, diameter: 10–20 nm, length: 10–30 μm, Nanjing Xianfeng Nano-technology Co., Ltd.), a PDMS base (10 g), and a curing agent (1 g) (with a weight ratio of 10:1, Sylgard 184, Dow Corning Co., Ltd) were dispersed in trichloromethane (265 g, Aladdin, 99%) for 2 h with ultrasonic assistance. For each layer, the solution was then poured into a mold, the trichloromethane was allowed to evaporate at room temperature, and the degassed liquid was cured at 80 °C for 2 h. The flat electrode was controlled to be ~50 μm thick with a CNTs-to-PDMS weight ratio of 7 wt%. Similarly, by controlling the casted weight and area of the mold, the dielectric layer was controlled to be 120 μm thick with a CNTs-to-PDMS weight ratio of 2 wt%.

*Calathea zebrine* leaves were used as the template for the fabrication of the microconed electrode. The *Calathea zebrine* leaves were washed clean and cut into rectangular pieces (area: 50 mm × 80 mm), which were then fixed onto glass substrates using pieces of Scotch tapes (3 M). A PDMS base and a curing agent (weight ratio of 5:1) were casted on the surface of the *Calathea zebrine* templates. After being cured at 70 °C for 1 h, the PDMS pieces with structures featuring microholes were peeled off and served as the secondary templates. Each PDMS template was then subjected to air plasma treatment (TS-PL05, Dongxingaoke Co., Ltd) at 50 W for 3 min. Finally, uncured PDMS-CNTs liquid was casted on the secondary template. After being cured at 80 °C for 2 h, the microstructured PDMS films featuring microcones were carefully peeled off and were ~100 μm thick with a CNT-to-PDMS weight ratio of 7 wt%.

### Interlinking of functional layers

After exploring the effect of different weight ratio of PDMS base and curing agent on the sensing performance (Supplementary Fig. 26), a weight ratio of 10:1 was chosen for the interlinking PDMS network. To introduce interlinks between the adherend networks, a PDMS base (5.5 wt%) and a curing agent (0.55 wt%) were added to trichloromethane and sonicated for 30 min to obtain a homogenized dispersion. The electrodes and the dielectric layer were then swollen in the trichloromethane solution for 6 h at room temperature. The swollen components were removed from the solution to allow the trichloromethane to evaporate. Finally, the flat electrode, the dielectric layer, and the microstructured electrode were stacked in order from top to bottom, exposed to a pressure of 20 kPa, and stored at 80 °C for 2 h for the monomers to be cured and interlinked.

### Sensing performance of the sensors

Most of sensors used for testing sensing properties have a surface area of 10 mm × 10 mm unless otherwise specified. To test of the sensitivity and the limit of detection, a sensor was placed on the flat stage of a mechanical test system (XLD-500E, Jingkong Mechanical Testing Co., Ltd). A flat indenter (larger than the sensor size) was used to slowly approach the sensor, make contact with the sensor, and load the programmed-set force. Capacitance signal was recorded in real-time during the loading process via an LCR meter (E4980AL, KEYSIGHT) at a testing frequency of 0.1 MHz. For the test of the response/release time, we used a general operation in the field^18,53^. First, a weight (5 g) was placed close to the top surface of the sensor (7 mm × 7 mm) and released carefully, producing a pressure of 1.1 kPa. Next, the weight was removed after placing for 5 s. The capacitance signal was recorded using an LCR meter (E4981A, KEYSIGHT) at a frequency of 1 MHz to obtain the response and recovery time.

### Stability testing

Sensors (10 mm × 20 mm in area) were adhered to specifically designed holders using a layer of cyanoacrylate glue (Krazy Glue, 3 M CA40H) to ensure that it could be fixed stably during test. The holders were controlled using a force gauge with a computer-controlled stage (XLD-500E, Jingkong Mechanical Testing Co., Ltd). For the rubbing tests, the pressure applied to each sensor was 10 kPa and the test speed was 2 mm·min^−1^ to realize a reciprocating displacement of 2 mm for 100,000 cycles. For the shear tests, the shear stress was 5 kPa for 10,000 cycles.

### Mechanical characterization

To measure interfacial toughness and shear strength, adhered samples, each with a surface area 10 mm in width and 30 mm in length, were prepared and subjected to a standard 180° peel test using a force gauge with a computer-controlled stage (XLD-500E, Jingkong Mechanical Testing Co., Ltd). The peeling speed was controlled at 50 mm·min^−1^. The sensors were adhered to a polyimide sheet by using a layer of cyanoacrylate glue (Krazy Glue) as a stiff backing.

### Testing of the sensor on a tire tread

The tire-tread sensing system consisted of a sensor (10 mm × 40 mm in surface area), a data acquisition module, and a data receiver. A commercialized flexible pressure sensor (MD30-60-50Kgf, diameter: 25 mm) was purchased from Suzhou Leanstar Electronic Technology Co., Ltd (website: http://www.lssensor.com) as a control sample to compare with our sensor in terms of sensing stability. The sensors were adhered to the tread of a rear tire on a car using VHB tape (3 M) and was connected to the data acquisition module attached to the wheel hub. Data was transmitted from the data acquisition module to the data receiver via Bluetooth. The driving conditions (including speed and distance) were recorded simultaneously through an GPS app on a phone. To avoid the complicated road condition, we chose to drive on a new asphalt loop-road (~300 m long) containing straight and turns.

### Sensor-soft robot integration and sensing performance during grasp manipulation

A pneumatic soft robot with three gripper fingers was used for sensor-robot integration. The grippers had a PDMS-CNTs (0.2 wt%) matrix and eight sensors (10 mm × 14 mm in area) were adhered on the gripper surface with interlinked interface by using the aforementioned methods. A netted melon (weight: 1250 g) and a doll (weight: 180 g) were chosen as specific subjects for the grasp manipulation. The driving pressure for griping the netted melon and the doll were 140 kPa and 80 kPa, respectively. For the bimodal sensing test, the resistance signals were collected from the flat electrode.

### Characterization

A field-emission scanning electron microscope (FESEM, TESCAN) was used to characterize the morphology of the sensor structures and the interfaces between the robot and the sensor. External pressure was applied and measured using a force gauge with a computer-controlled stage (XLD-500E, Jingkong Mechanical Testing Co., Ltd). The capacitance response and the permittivity were measured via an LCR meter (E4980AL, KEYSIGHT) at a testing frequency of 0.1 MHz unless otherwise specified. The response time and relaxation time were measured via an LCR meter (E4981A, KEYSIGHT) at a testing frequency of 1 MHz. The resistance was recorded using a digital multimeter (Keithley 2100). For the stable electrical connection of the sensor to the LCR meter, two thin silver wires (dimeter ~0.1 mm) were adhered on a protruding part of the electrodes by using soft elargol, and a thin PDMS layer was further casted and cured to protect the joint.

### Reporting summary

Further information on research design is available in the Nature Research Reporting Summary linked to this article.

## Supplementary information

Supplementary Information

Description of Additional Supplementary Information

Supplementary Movie 1

Supplementary Movie 2

Reporting Summary

## Data Availability

All relevant data sets generated during and/or analyzed during the current study are available from the corresponding author upon reasonable request.
