# Supplementary material for: Highly stable flexible pressure sensors with a quasi-homogeneous composition and interlinked interfaces
Source: Nat Commun. 2022 Mar 10;13:1317. doi: 10.1038/s41467-022-29093-y (PMC8913661; doi:10.1038/s41467-022-29093-y)
Supplement: Supplementary file 1 — Supplementary Information [file 41467_2022_29093_MOESM1_ESM.pdf]

Supporting Information

Highly stable flexible pressure sensors with a quasi-homogeneous composition and interlinked interfaces

Yuan Zhang[1,#], Junlong Yang[2,#], Xingyu Hou[1], Gang Li[1], Liu Wang[1], Ningning Bai[1], Minkun Cai[1], Lingyu Zhao[1], Yan Wang[1], Jianming Zhang[1], Ke Chen[3], Xiang Wu[4], Canhui Yang[5], Yuan Dai[3], Zhengyou Zhang[3], and Chuan Fei Guo[1,5,*]

[1]Department of Materials Science and Engineering, Southern University of Science and Technology, Shenzhen Guangdong 518055, China

[2]College of Polymer Science and Engineering, State Key Laboratory of Polymer Materials Engineering of China, Sichuan University, Chengdu, 610065, China

[3]Tencent Robotics X, Shenzhen Guangdong 518000, China

[4]School of Materials Science and Engineering, Shenyang University of Technology, Shenyang 110870, China

[5]Department of Mechanics and Aerospace Engineering, Southern University of Science and Technology, Shenzhen Guangdong 518055, China

[#] These authors contributed equally to this work.

*Correspondence and requests for materials should be addressed to C. F. Guo (email: guocf@sustech.edu.cn)

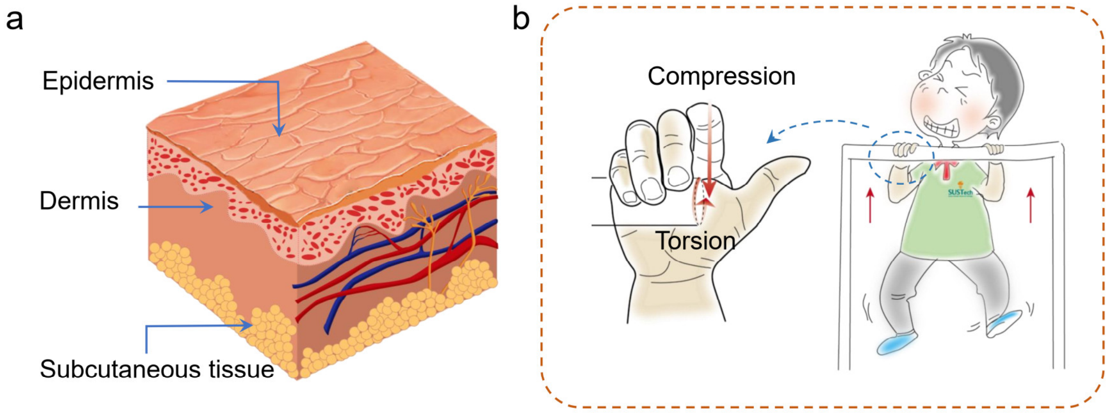

Supplementary Figure 1 | Schematic illustrations of human skin and its force analysis under loading. a) The skin shows a layered structure consisting of the epidermis, the dermis, and the subcutaneous fat tissue. b) Human skin is simultaneously subjected to compression and torsion under certain conditions such as while performing a pull-up exercise.

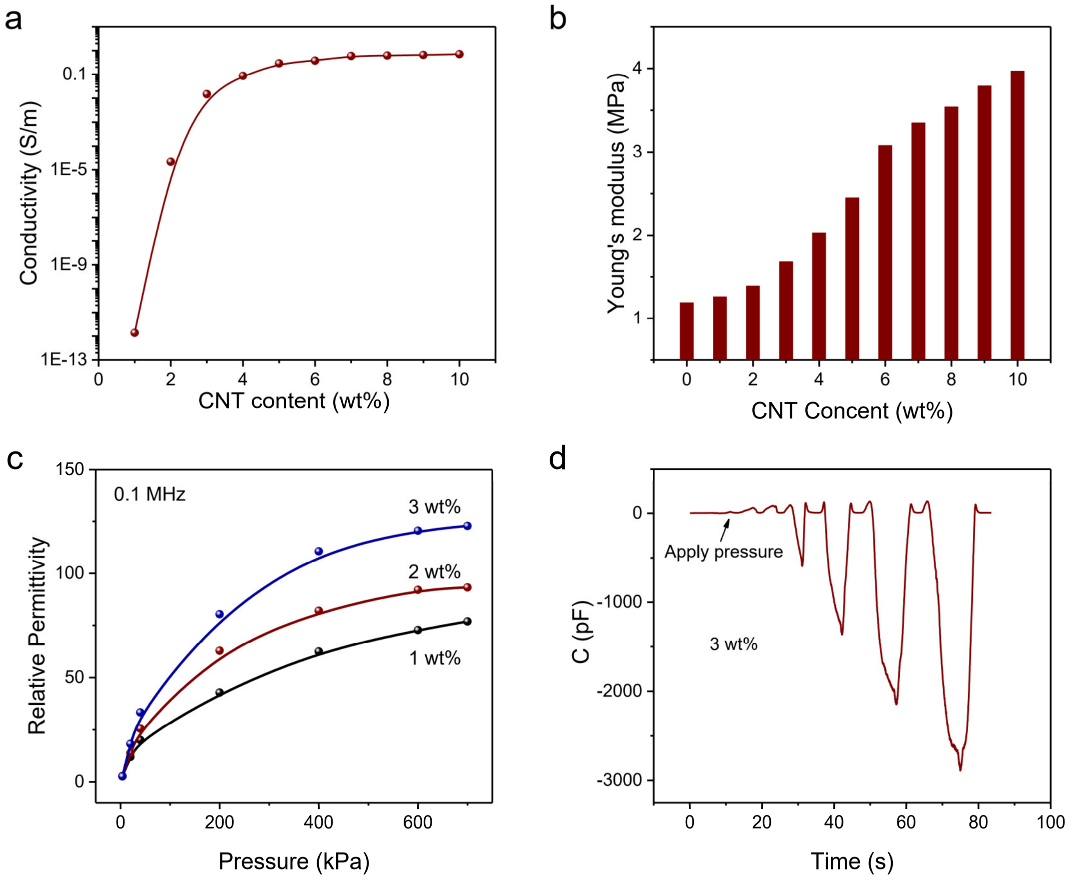

Supplementary Figure 2 | Properties of the PDMS-CNTs composites. a) Electrical conductivity and b) Young's modulus of the PDMS-CNTs composites as a function of CNT content. c) Relative permittivity of the PDMS-CNTs composites with 1, 2, and 3 wt% CNTs under different pressures. d) Negative response of a sensor that uses the PDMS-CNTs (3 wt%) composite as the dielectric layer.

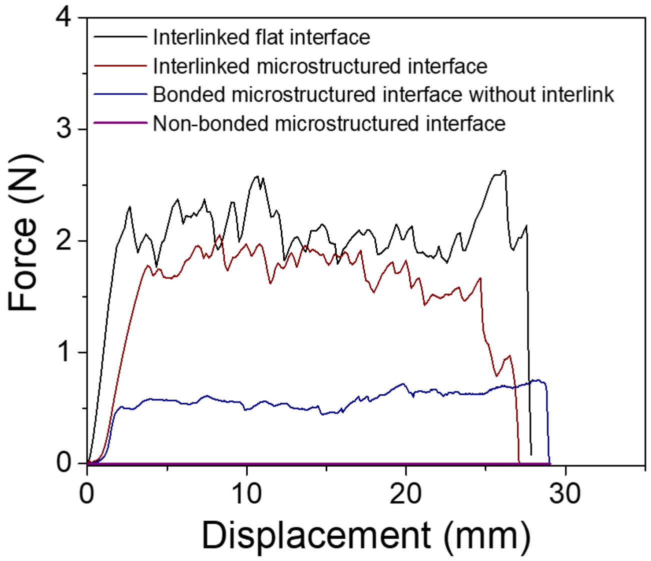

Supplementary Figure 3 | The force-displacement curves of samples with different interfaces during 180° peel test.

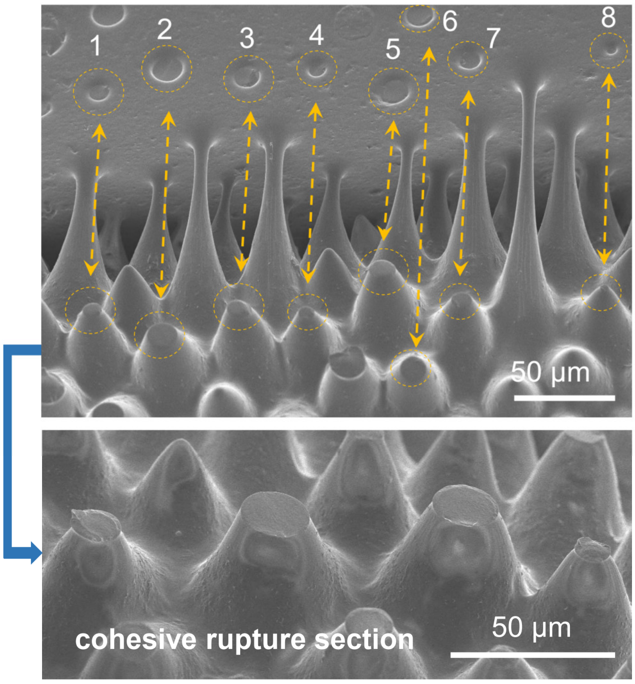

Supplementary Figure 4 | SEM images of the microstructured interface under peeling, showing cohesive rupture of the microcones. The upper panel shows 8 ruptured microcones, as well as a few short cones that had not bonded to the dielectric layer and retain a complete cone shape. The lower panel shows the detailed morphology of the fracture.

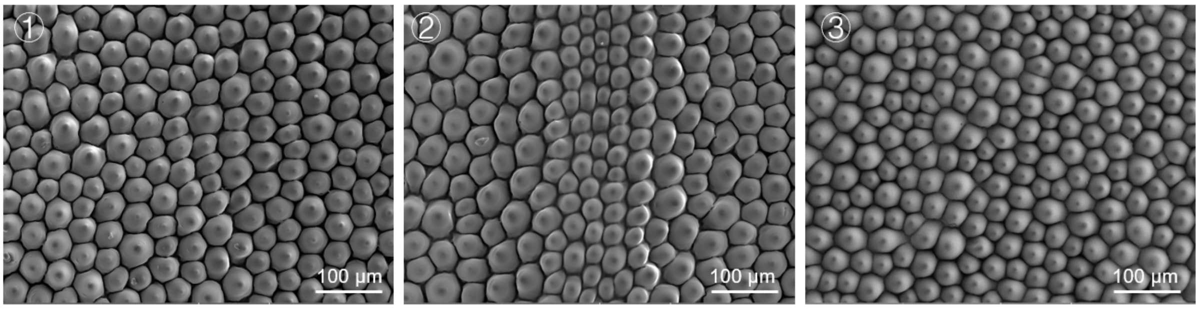

Supplementary Figure 5 | SEM images of the spatial distribution of the cones viewed from different regions. The average density of the cones is about 7.9×10^{8} m^{-2}. The density of the cones from the first region to the third region is about 7.8×10^{8} m^{-2}, 7.9×10^{8} m^{-2} and 8.0×10^{8} m^{-2}, respectively.

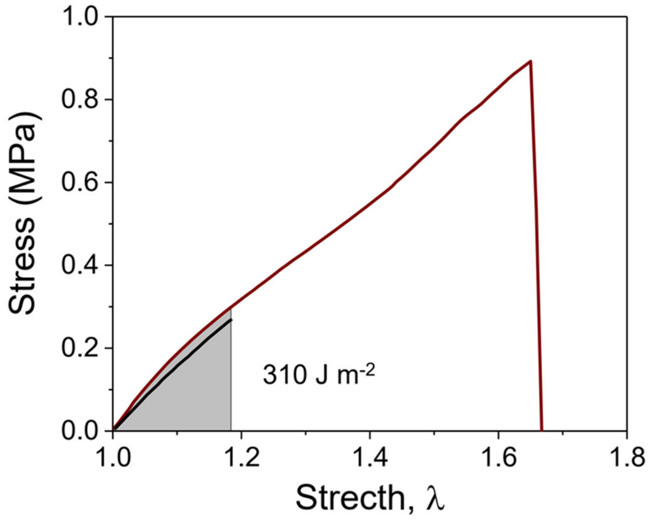

Supplementary Figure 6 | Fracture toughness of pure PDMS. Each sample has a length of 50 mm, a thickness of 0.8 mm, and a height of 10 mm. The loading rate was 50 mm min^{-1}.

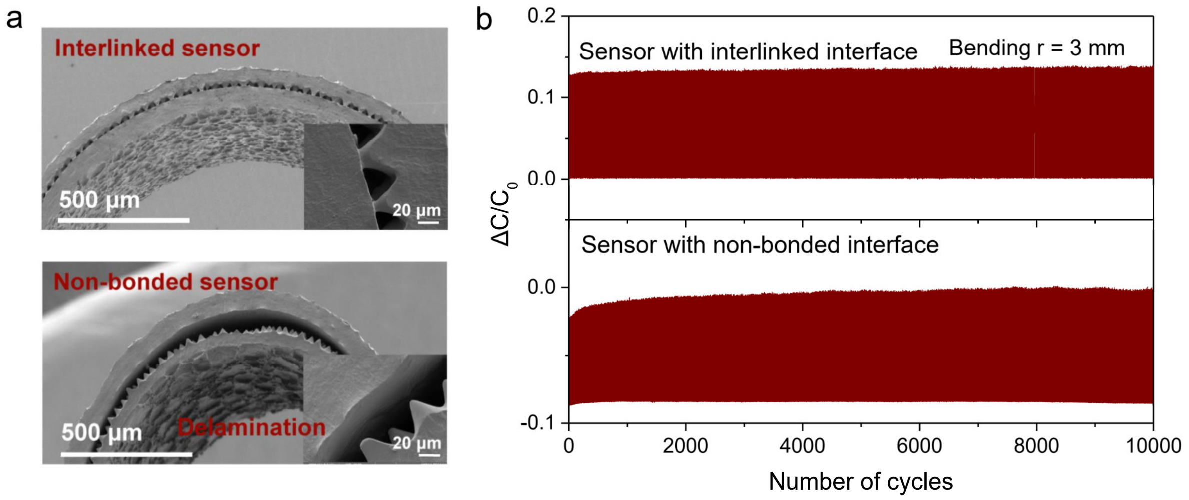

Supplementary Figure 7 | Stability of sensors under bending. a) SEM images of sensors with and without a bonded interface under bending (upper and lower panels, respectively), in which the non-bonded sensor shows layer delamination. b) Normalized capacitance changes of each of the two sensors under cyclic bending with a bending radius of 3 mm. The sensor with an interlinked interface shows higher signal stability.

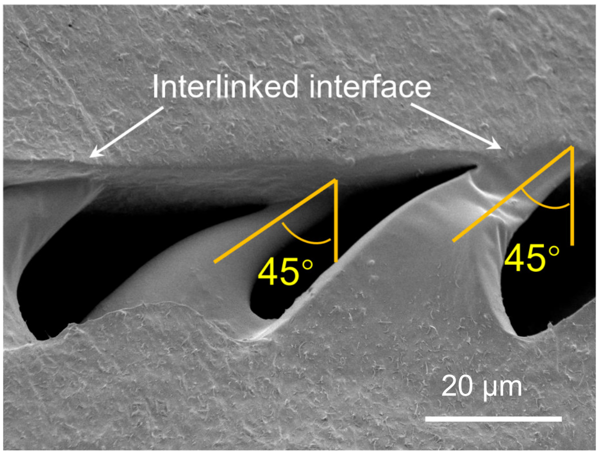

Supplementary Figure 8 | Shear strain of the microstructured interface under twisting. SEM image shows that interlinked interface is under a large shear strain of ~0.8 (or 45°), and the cones remain firmly bonded to the dielectric layer at such a high shear strain.

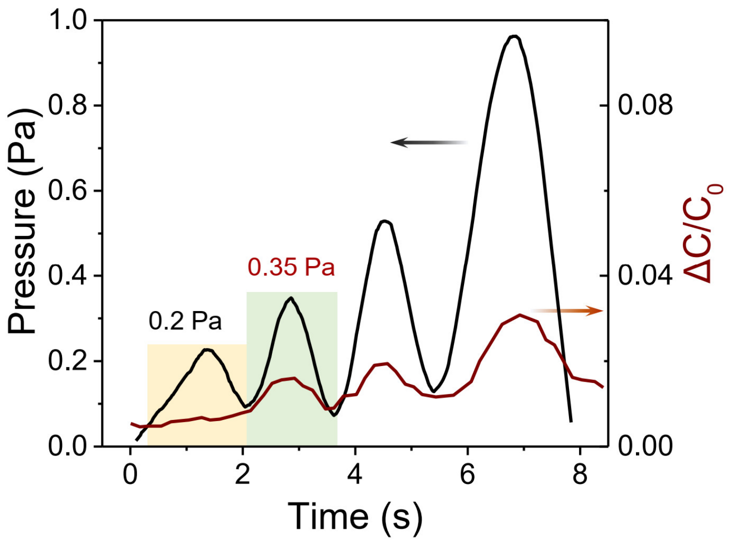

Supplementary Figure 9 | The limit of detection (LOD) test of the pressure sensor.

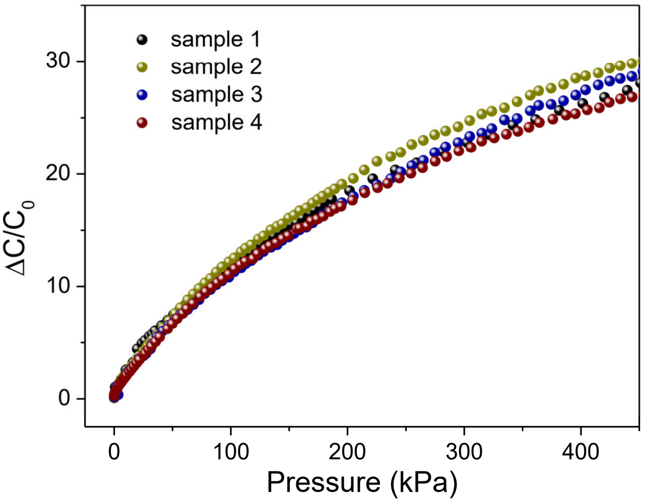

Supplementary Figure 10 | Normalized capacitance changes of four sensors from different batches, showing a reasonable repeatability.

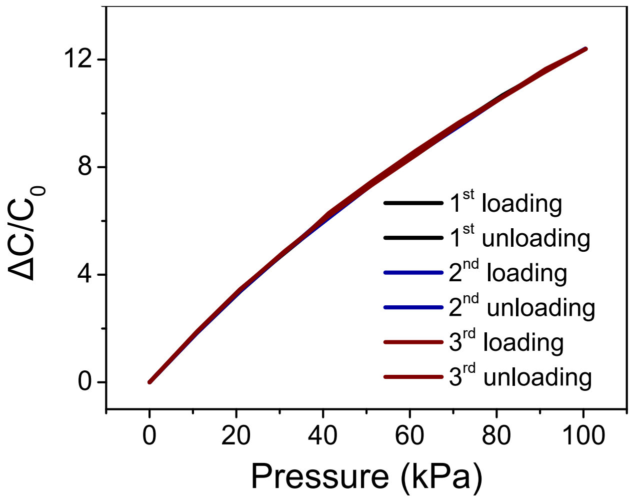

Supplementary Figure 11 | Hysteresis loops of loading to 100 kPa and unloading over three cycles.

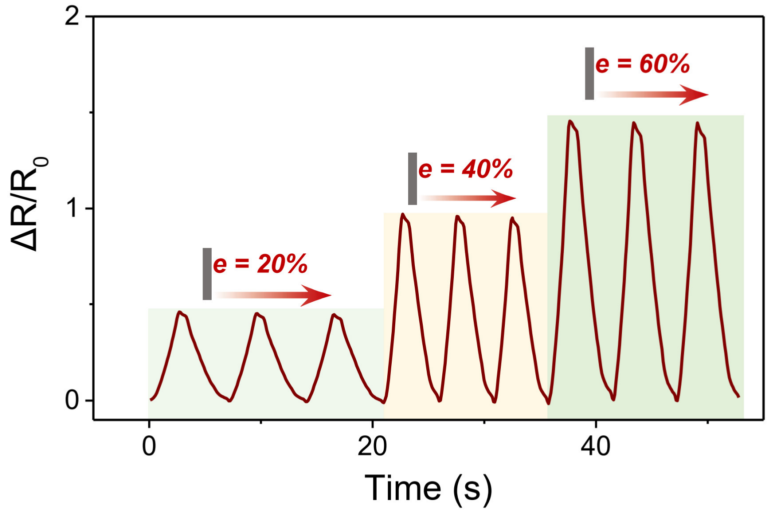

Supplementary Figure 12 | Normalized change in resistance of the PDMS-CNTs electrode (7 wt% CNTs) under different levels of strains of 20%, 40%, and 60% over a few cycles at each level.

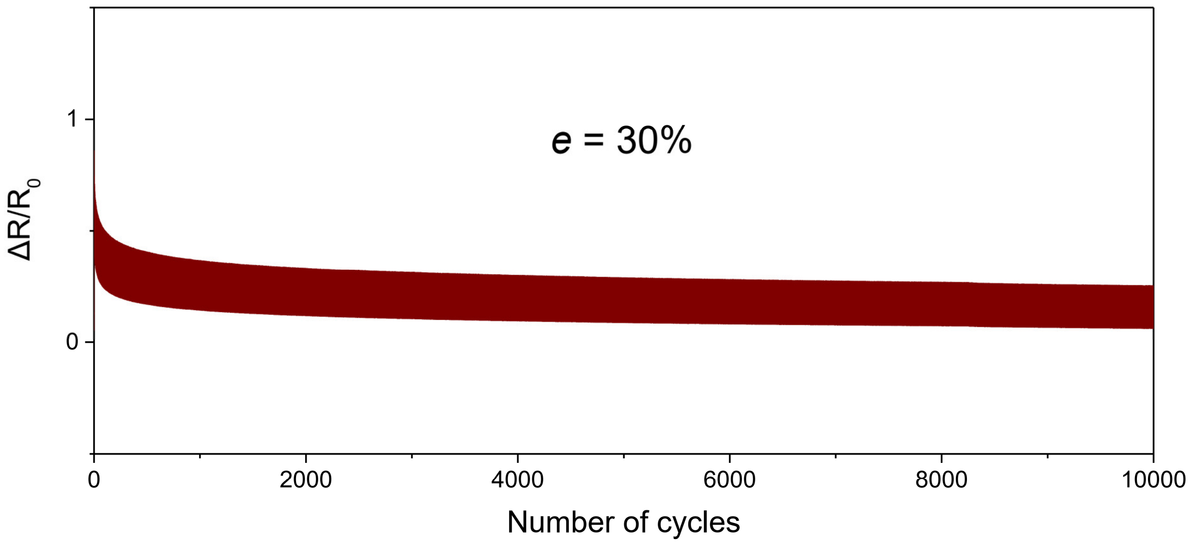

Supplementary Figure 13 | Normalized change in resistance of the PDMS-CNTs electrode (7 wt% CNTs) stretched to a strain of 30% over 10,000 cycles. Note that there is a decrease of the baseline at the beginning of the test. For real applications, the pressure sensor is often pre-stretched for several times to ensure a stable response.

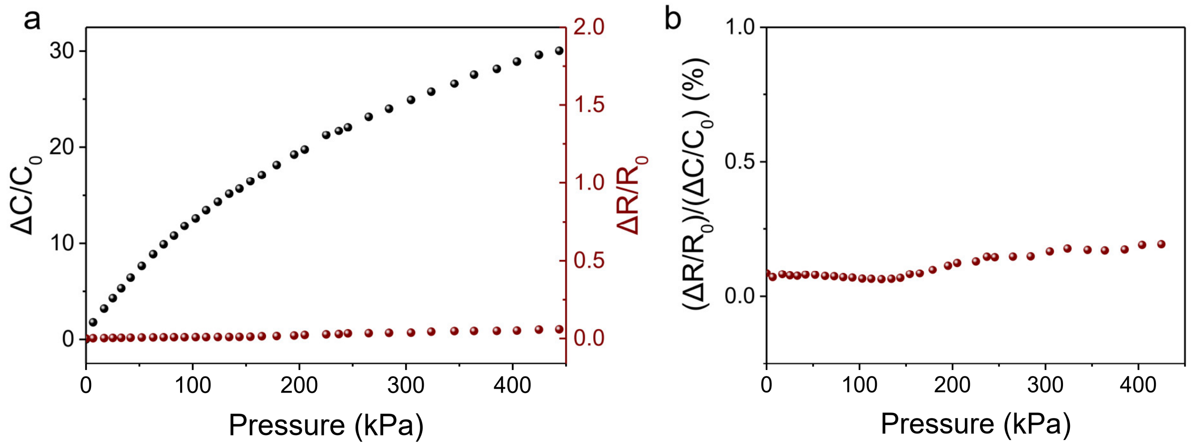

Supplementary Figure 14 | Normalized change in capacitance and resistance of the sensor as a function of pressure. a) The comparison of $\Delta C/C_0$ and $\Delta R/R_0$. b) The ratio between resistive signal and capacitive signal as a function of pressure, showing that the ratio of $(\Delta R/R_0)/(\Delta C/C_0)$ is below 0.2%. This indicates that the coupling of the two signals is negligible.

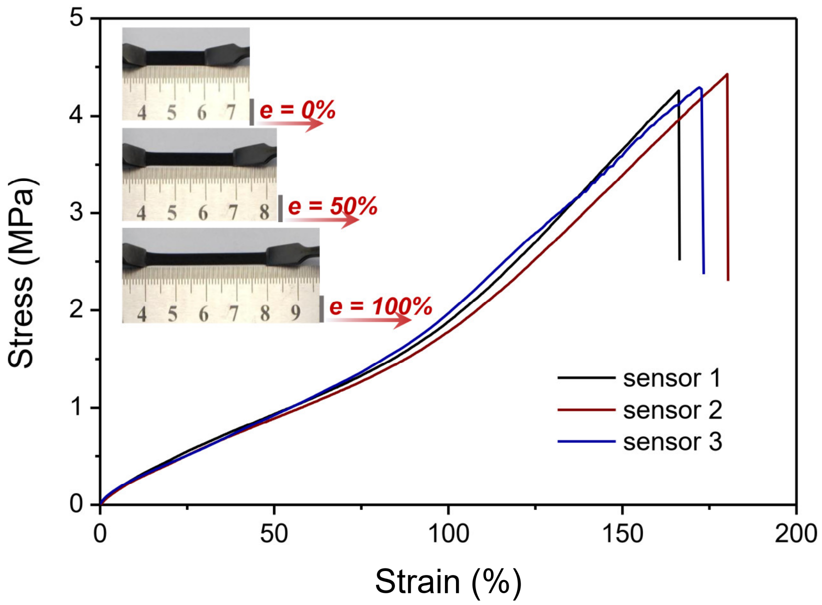

Supplementary Figure 15 | Stress-strain curves of three PDMS-CNTs sensors. It shows a large stretchability of at least ~160%. Photographs of the sensor under different levels of strains are shown in the insets.

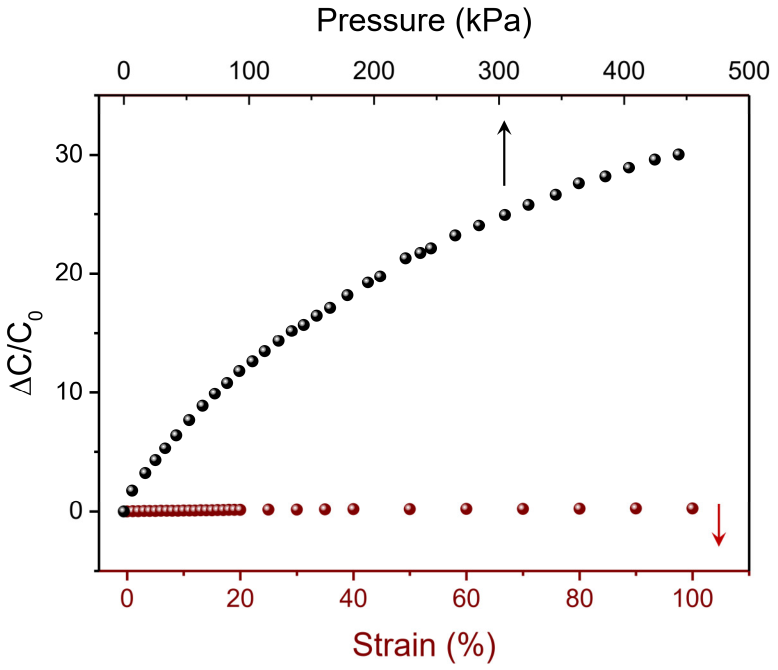

Supplementary Figure 16 | Normalized capacitance change of the flexible sensor under different strains (up to strain of 100%) and pressures (up to 450 kPa).

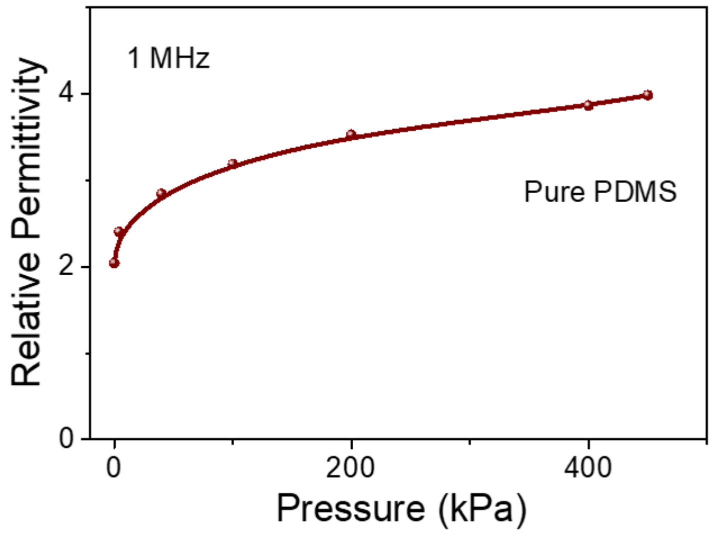

Supplementary Figure 17 | The relative permittivity of pure PDMS under different pressure.

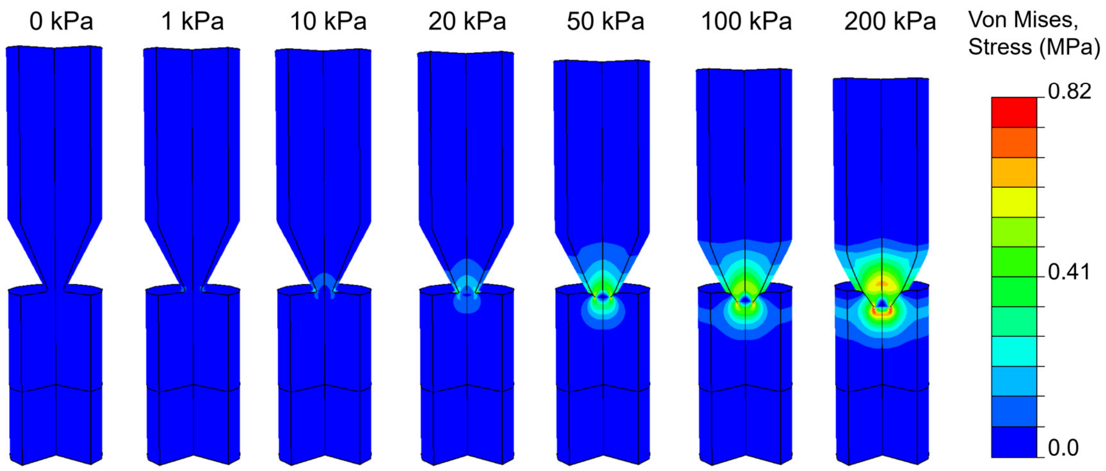

Supplementary Figure 18 | Finite element modeling of stress distribution of a microcone under applied pressure up to 200 kPa. Finite element simulations were conducted using a commercial package Abaqus/Standard 2017. The electrode, dielectric layer, and surface microcone are modeled as incompressible materials with Young's moduli of 3.4 MPa, 1.4 MPa, and 1.4 MPa, respectively. An axisymmetric model of a surface microcone was built and compressed against the dielectric layer under pressure up to 200 kPa.

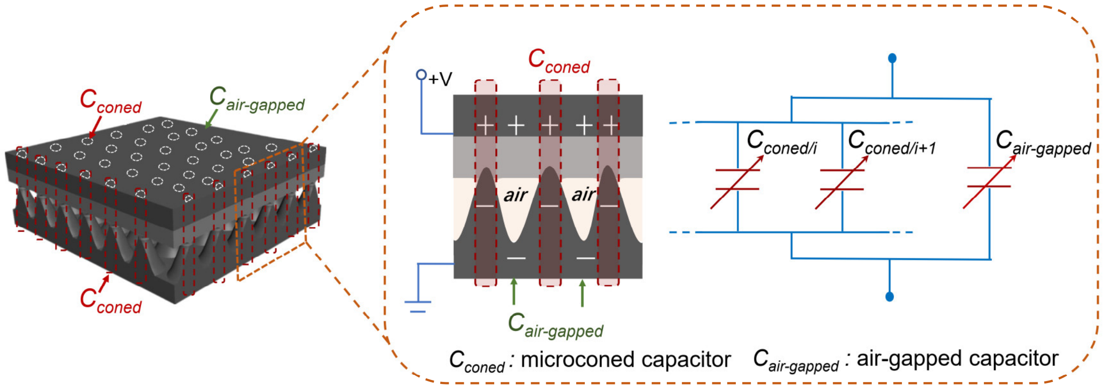

Supplementary Figure 19 | Schematic illustration of the sensor structure. The pressure sensor consists of many microconed capacitors and a mesh-like air-gapped capacitor connected in parallel to offer capacitance changes under stimuli. Upon loading, the microconed capacitors can have a significant change in capacitance, while the latter contributes much less than the microconed capacitors.

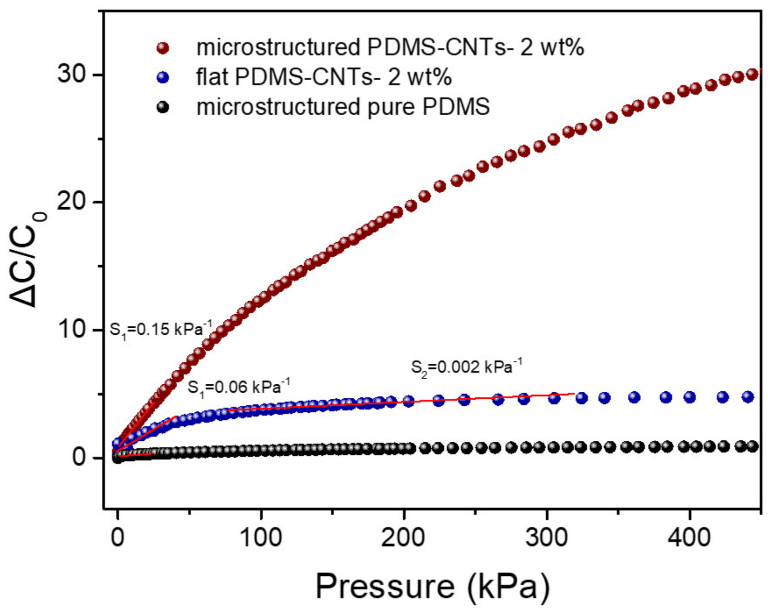

Supplementary Figure 20 | Normalized change in capacitance as a function of pressure of sensors with different dielectric layers: microstructured PDMS-CNTs composite, flat PDMS-CNTs composite, and microstructured pure PDMS. Both the sensor with a flat PDMS-CNTs dielectric and that with a microstructured pure PDMS dielectric show relatively low response and narrow pressure response range, while the sensor with microconed PDMS-CNTs dielectric exhibits much stronger response and a wider pressure response range.

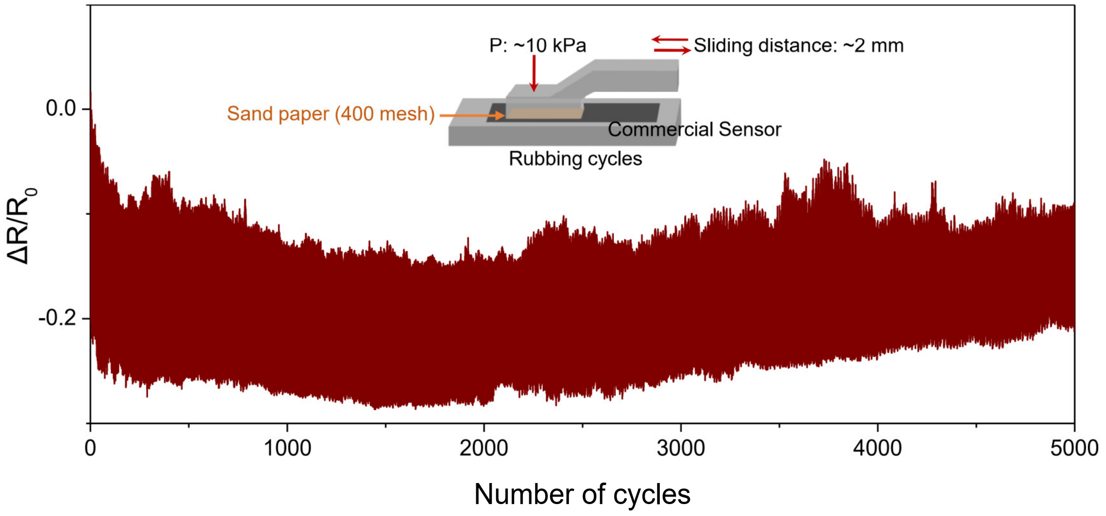

Supplementary Figure 21 | Normalized change in resistance of the commercial sensor under cyclic rubbing. Inset: schematic illustration of the rubbing test, which was setup with the same test condition as described in Figure 3g.

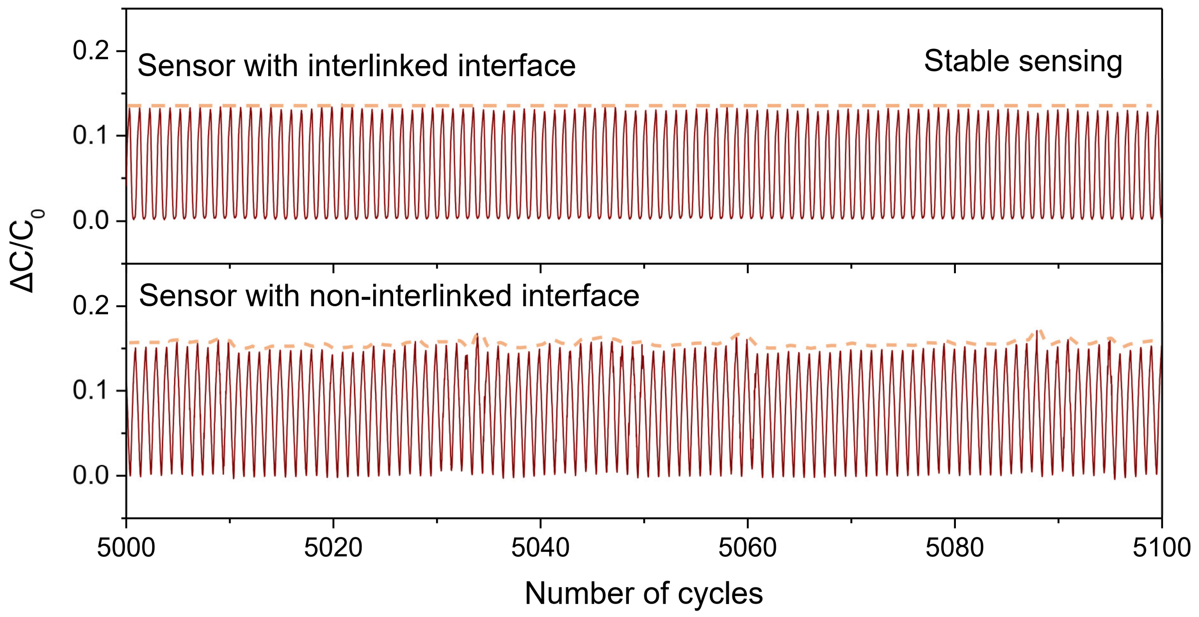

Supplementary Figure 22 | Comparison of capacitance changes between sensors with and without interlinked interfaces (upper and lower panels, respectively) over the 5000th-5100th cycles under repeated shear stress of 5 kPa.

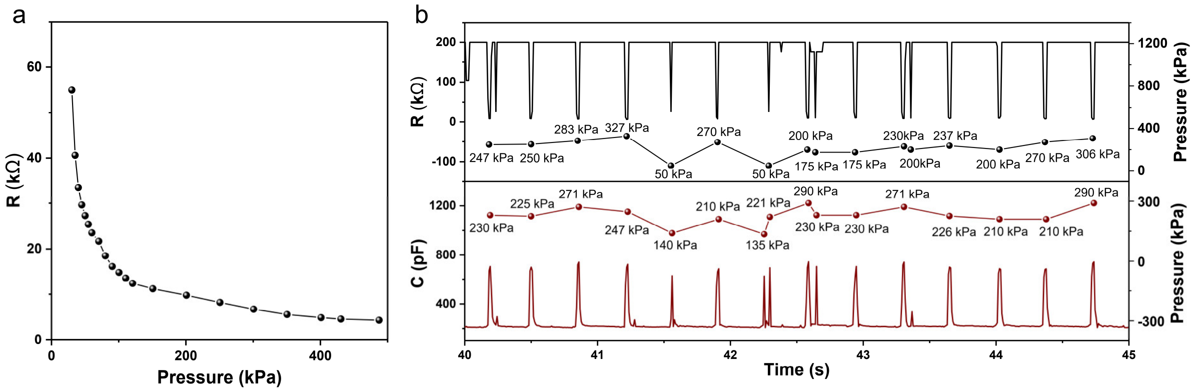

Supplementary Figure 23 | a) Change in resistance as a function of pressure of the commercial sensor. b) Real-time sensing signals, and the pressure values measured using the commercial sensor and our sensor at the beginning of the driving test (40-45 s). The resistance signal is for the commercial sensor and the capacitance signal is for our sensor.

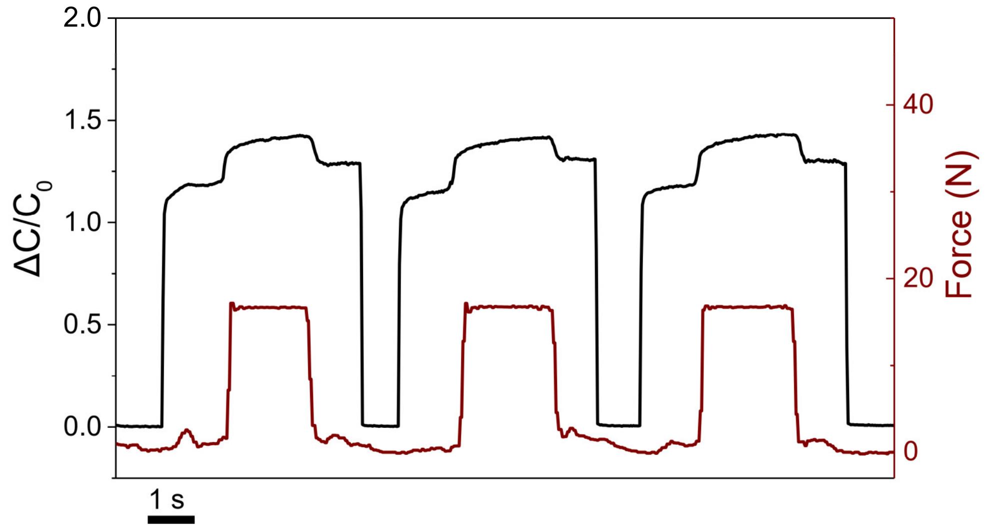

Supplementary Figure 24 | Capacitance change and corresponding force applied to lift the soft robot during cyclic gripping and release of the melon.

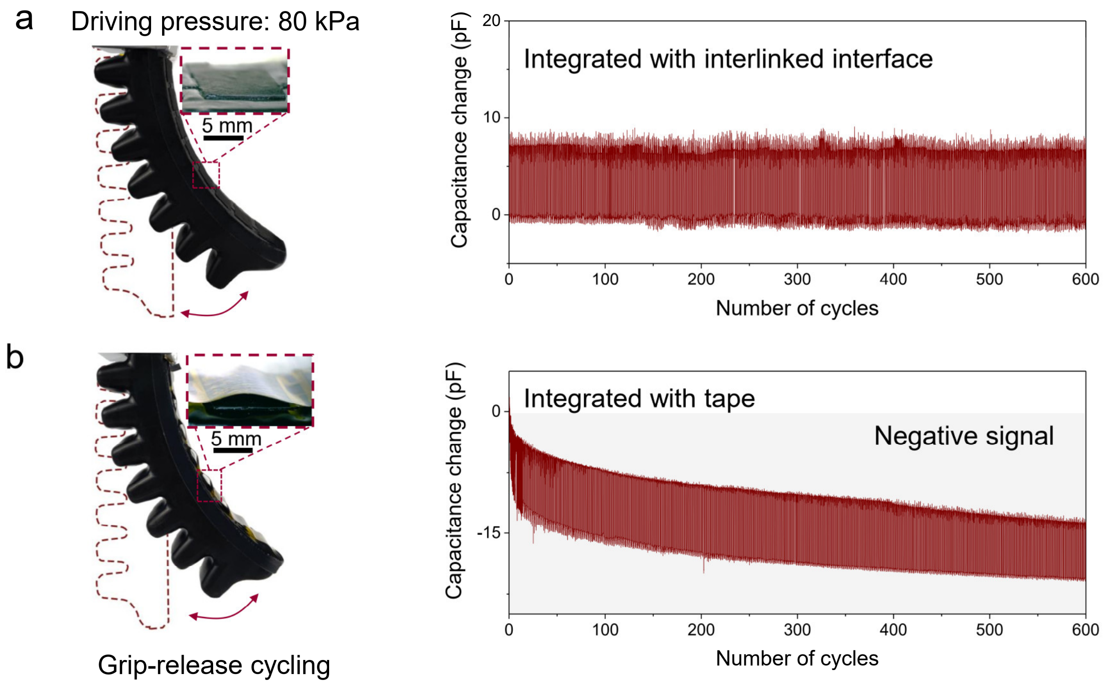

Supplementary Figure 25 | The stability of sensors upon bending of the soft-robot gripper. a) Appearance of the sensor with interlinked sensor-robot interfaces, and corresponding capacitance change over 600 grasp-release cycles for a sensor integrated on of soft-robot gripper. b) Appearance of non-bonded sensor that is encapsulated with tapes, and corresponding capacitance change under 600 grasp-release cycles. Insets to the schematic illustrations in panels (a) and (b) show optical images of the sensors integrated with an interlinked interface and with tape, respectively.

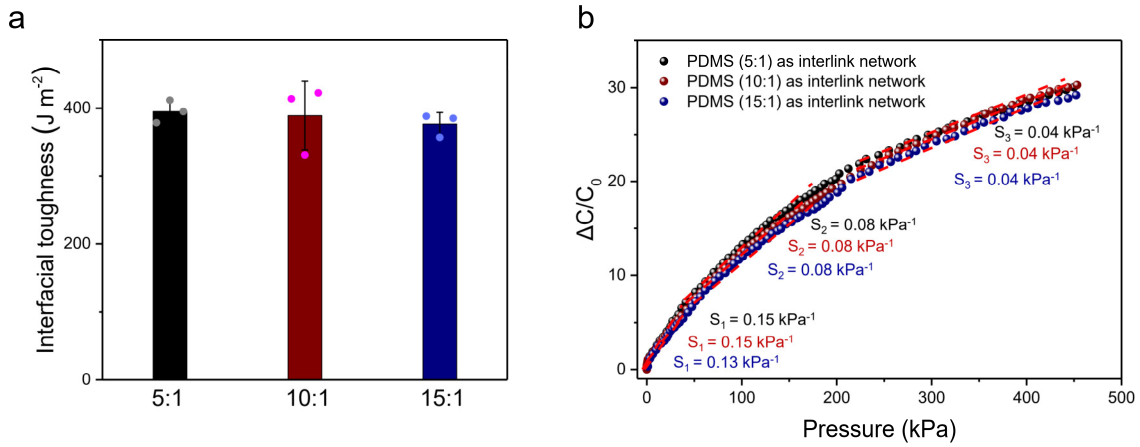

Supplementary Figure 26 | a) Interfacial toughness between the dielectric layer and the microstructured electrode using PDMS (base to curing agent ratio of 5:1, 10:1, and 15:1) as the interlink network. b) Normalized capacitance changes of three sensors using PDMS-CNTs dielectric with base to curing agent ratios of 5:1, 10:1 and 15:1 as the interlink network.

Supplementary Note 1 | Experimental setup details for the car driving demonstration. Each sensor used is 10 mm × 40 mm in surface area and was adhered to the tread of a rear tire using VHB tape (3M). The sensing signal was recorded using a wireless system that consists of a data acquisition module and a data receiver. The driving conditions (including speed and distance) were simultaneously recorded using a Global Positioning System (GPS) application on a smartphone. To avoid complicated road conditions, the test car was driven on a new asphalt loop-road (~300 m) with both straightaways and turns.

Supplementary Note 2 | Calculation of the shear stress for the sensor during car driving. The shear stress is determined by the pressure (P) applied on the tire-road interface and the rolling resistance coefficient f. That is, shear stress equals to $P \cdot f$. The mass of the car is about 2000 kg, and its weight is shared by four tires (suppose a uniform distribution). The tire-road contact area for each tire is determined to be ~160 cm^2, and thus the pressure is ~300 kPa. Typically, the f value for an asphalt road is about 0.02, and the estimated shear stress is determined to be ~6 kPa.
