# Supplementary material for: Highly stable flexible pressure sensors with a quasi-homogeneous composition and interlinked interfaces
Source: Nat Commun. 2022 Mar 10;13:1317. doi: 10.1038/s41467-022-29093-y (PMC8913661; doi:10.1038/s41467-022-29093-y)
Supplement: Supplementary file 2 — Description of Additional Supplementary Information [file 41467_2022_29093_MOESM2_ESM.pdf]

Description of Additional Supplementary Information

Title: Supplementary Movie 1

Description: Finite element modeling results of the stress distribution in the dielectric and the cone upon loading

Title: Supplementary Movie 2

Description: Output of the sensor attached on a tire tread when the car drives on an asphalt road.
